# Microwave-Assisted Bio-Based Chemical Recycling of Fiber-Reinforced Composites from Construction and Demolition Waste

**DOI:** 10.3390/polym18030362

**Published:** 2026-01-29

**Authors:** Gonzalo Murillo-Ciordia, Cecilia Chaine

**Affiliations:** CIRCE—Technology Centre for Energy Resources and Consumption, Parque Empresarial Dinamiza, Av. Ranillas 3D, 1st Floor, 50018 Zaragoza, Spain

**Keywords:** microwave-assisted solvolysis, green solvents, fiber-reinforced polymer composites, construction and demolition waste

## Abstract

Fiber-reinforced polymer composites (FRPCs) are increasingly used in construction due to their high performance and low environmental footprint. However, their widespread adoption has raised concerns over end-of-life management, particularly under European regulations mandating high recycling rates for construction and demolition waste (CDW). This study evaluates different systems for the chemical recycling of FRPCs through microwave (MW)-assisted solvolysis using green solvents, including deep eutectic solvents (DESs) and biobased acetic acid. The process targets thermoset resin depolymerization while preserving fiber integrity, operating at reduced temperatures (≤230 °C) and lower energy demand than conventional techniques, such as pyrolysis. A systematic experimental design was applied to CDW-derived polyester composites and extended to industrial epoxy and vinyl ester composites. Among the tested solvents, glacial acetic acid + ZnCl_2_ (5 wt.%), achieved the highest degradation efficiency, exceeding 94% in small-scale trials and maintaining over 78% upon upscaling. Recovered fibers showed moderate property retention, with tensile strength and elongation losses of ~30% and ~45% for infusion-based epoxy composites, while those from pultrusion-based epoxy composites exhibited 16–19% and retained similar properties to the virgin material, respectively. The method facilitates fiber recovery with limited degradation and aligns with circular economy principles through solvent reuse and minimizing environmental impact.

## 1. Introduction

The increasing legislative pressure on waste management within Europe underscores the need for innovative valorization pathways for construction and demolition waste (CDW), which is estimated to account for 25–30 percent in weight of total waste generated in the EU [1]. In response to this regulatory context, Spain has recently adopted the State Waste Management Framework Plan (PEMAR 2024–2035), approved in January 2025 (BOE-A-2025-1118), which defines measurable national objectives for waste prevention and management in line with Directive 2008/98/EC. Specifically, the plan targets that at least 75% by weight of non-hazardous CDW is to be reused, recycled, and other recovery operations are to be developed by 2035.

Fiber-reinforced polymer composites (FRPCs) are widely used across construction, energy, transport, and marine sectors due to their strength-to-weight ratio, durability, and corrosion resistance. However, their thermoset-based matrices (i.e., epoxy, vinyl ester, and polyester resins) are cross-linked and consequently non-meltable and difficult to recycle. Thus, the rapid growth in FRPC use has generated an emerging waste stream lacking efficient end-of-life solutions [2,3].

Chemical recycling, in particular solvolysis, has gained attention as a means to recover fibers and potentially reuse the polymeric fraction by depolymerizing the matrix in reactive solvent under moderate conditions (generally <350 °C) [4]. Despite its advantages, conventional solvolysis is energy-intensive (21–91 MJ/kg) and often relies on hazardous chemicals [5]. To achieve truly sustainable recycling, the process must therefore minimize both its environmental impact and energy demands.

In this context, the use of green solvents, such as ionic liquids (ILs), deep eutectic solvents (DESs), and bio-based solvents, offers a promising pathway. These solvent systems combine high selectivity and mild operating conditions with low volatility. Furthermore, DESs and bio-based solvents are biodegradable and easy to prepare [6,7,8,9]. Several studies have demonstrated the ability of such solvents to selectively degrade thermoset matrices while maintaining fiber integrity, indicating their suitability for circular recycling approaches [7,9]. Microwave (MW)-assisted heating has emerged as a complementary technique that can enhance solvolytic reactions by providing fast, volumetric, and selective energy transfer to polar reaction media [10,11,12]. Compared with conventional heating, MW-assisted processes typically exhibit faster reaction rates and improved energy efficiency, making them attractive for chemical recycling applications [13,14]. However, the interaction between MW energy and non-conventional green solvents, such as DESs or bio-based solvents, remains insufficiently explored, and reliable data on optimal operating conditions for composite waste treatment are scarce [15,16,17,18]. While the present study does not resolve the fundamental MW–DES interaction mechanisms, it provides comparative experimental evidence on the solvolysis performance of DESs under MW conditions relative to bio-based and conventional solvents. To address this practical gap, we developed and evaluated an MW-assisted green solvolysis process for recycling CDW-derived FRPCs. A set of solvent systems, including DESs and bio-based media, was assessed to determine their efficiency, selectivity, and influence of operating conditions on fiber recovery and resin degradation. The resulting data contribute to defining environmentally responsible and potentially scalable chemical recycling pathways for composite waste management, while also laying out practical performance boundaries that can guide future research.

## 2. Materials and Methods

### 2.1. Materials

**Figure 1:**
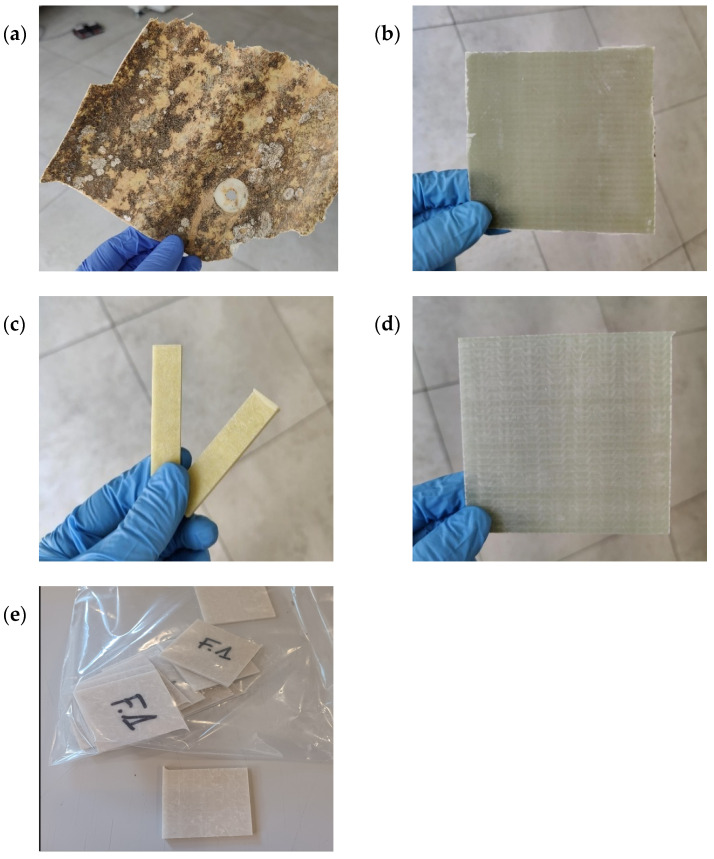
Fiber-reinforced polymer composites (FRPCs) investigated in this study: (**a**) CDW manager’s FRPC sample and manufacturer’s FRPC sample, (**b**) epoxy infusion (EP-IN), (**c**) epoxy pultrusion (EP-PU), (**d**) vinyl ester infusion (VE-IN) and (**e**) polyester pultrusion (EST-PU).

Five different composite samples were investigated in the present study (see Figure 1). One post-consumer material, supplied by a local CDW manager (Spain) originated from the dismantling of a building roof that had been exposed to environmental degradation factors, such as sunlight, humidity, and temperature variations. This real-waste sample was used to optimize the experimental solvolysis conditions. Once these were established, four construction composites, consisting of pre-consumer materials provided by a composite manufacturer (Spain) were tested to evaluate process applicability.

The post-consumer composite sample used in this study was sourced from a regional CDW manager in Aragón (Spain). Although the exact origin and service history of the component are unknown, reflecting a common situation in CDW, this lack of traceability is itself representative of the real waste treated in demolition and recycling facilities across Europe. Composites reaching CDW plants typically originate from roofing, façade elements, or structural profiles, and have been exposed for extended periods to the climatic conditions of the region, including high UV levels, temperature fluctuations, and humidity typical of the Spanish continental climate. The study therefore intentionally incorporates a sample collected from a real post-consumer waste stream. This heterogeneous FRPC captures the variability, degradation state, and information gaps that recycling technologies must handle.

In parallel, the four pre-consumer composites supplied by an industrial manufacturer were included to represent the principal resin chemistries and production routes used in European construction and infrastructure applications. Epoxy, polyester, and vinyl ester thermosets, combined with infusion and pultrusion manufacturing processes, collectively account for the majority of glass fiber composites used in panels, profiles, civil engineering components, and building elements. Although these specific items entered the study as waste offcuts, their resin–fiber architecture corresponds to standard commercial composite categories and therefore provides controlled, well-defined reference systems against which reaction mechanisms and fiber preservation can be reliably compared.

By pairing an aged, heterogeneous CDW sample with industrially relevant pre-consumer composites of known composition, the study evaluates both real-waste behavior and controlled, comparable reference systems. This two-tier material selection ensures that the developed process targets the most representative composites found at end-of-life, while remaining broadly applicable across the main resin types and manufacturing technologies used in the construction sector.

For experiments at the 40 mL scale, 3 g of material was used; CDW samples were washed and manually ground, while manufacturer samples were cut into fragments smaller than 5 × 5 cm to enhance solvent exposure and reproducibility. For the 600 mL-scale experiments, 45 g of material was used; CDW samples were cut into 10 cm-long pieces and manufacturer samples into 2 × 10 cm rectangles.

All reagents were used as received without further purification. Glacial acetic acid (≥99.7% purity, technical grade, VWR International S.A.S., Rosny-sous-Bois, France), ethylene glycol (≥99%, technical grade, VWR International S.A.S., Rosny-sous-Bois, France), zinc chloride (≥98%, VWR International S.A.S., Rosny-sous-Bois, France), choline chloride (≥98%, Sigma-Aldrich Co., St. Louis, MO, USA), urea (≥99%, EssentQ^®,^ Merck KGaA, Darmstadt, Germany), and glycerol (99%, Pharmapur^®^, Merck KGaA, Darmstadt, Germany) were used in the preparation of all solvent systems. All solvents and reagents met the purity levels commonly required for solvolysis and polymer degradation studies, ensuring reproducibility and comparability with the existing literature. Deep eutectic solvents (DESs) were prepared from these components as biodegradable alternatives for resin depolymerization.

### 2.2. Experimental Setup

**Figure 2:**
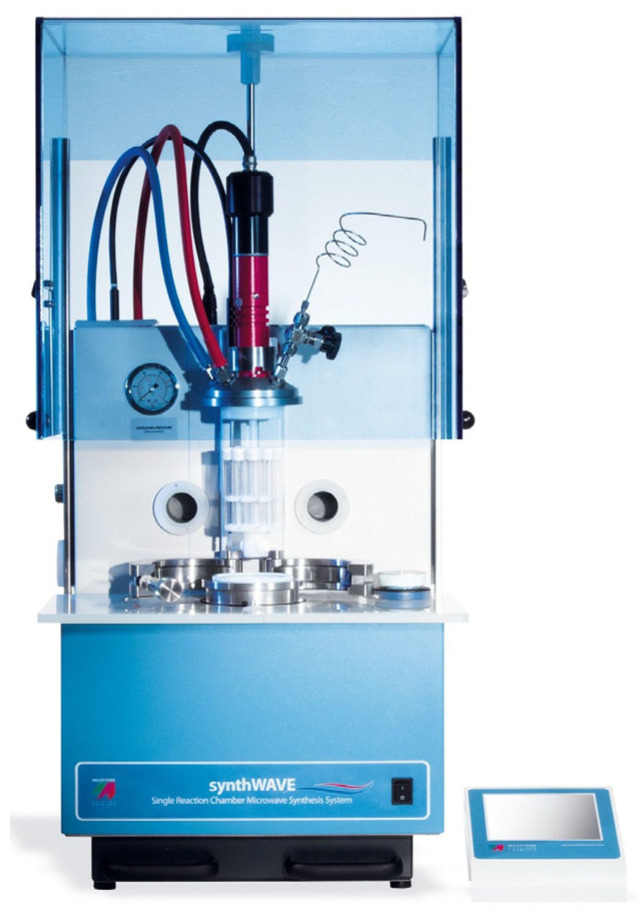
Milestone synthWAVE MW-assisted reactor (source: https://www.milestonesrl.com/es/productos/sintesis-por-microondas/synthwave, accessed on 27 January 2026).

Experimental trials were performed using an MW-assisted reactor (synthWAVE, Milestone, Sorisole, Italy—Figure 2) equipped with a 1 L Teflon-lined cylindrical reaction chamber. The system is capable of delivering up to 1500 W of microwave power and operates under maximum conditions of 300 °C and 199 bar with process parameters controlled via a PLC interface. The reactor was connected to a chiller to maintain the magnetron temperature below 45 °C.

The selected reagents targeted chemical depolymerization of the polymer matrix while preserving fiber integrity. Deep eutectic solvents (DESs), such as choline chloride combined with urea or glycerol, were evaluated as biodegradable, cost-effective alternatives. Biobased acetic acid was also tested for its low toxicity and availability. In some experiments, catalytic additives such as ZnCl_2_ were introduced to accelerate depolymerization.

### 2.3. Desing of Experiments

The experimental plan aimed to develop an efficient and environmentally sound process for decomposing polymer resins in FRPCs via MW-assisted solvolysis.

**Figure 3:**
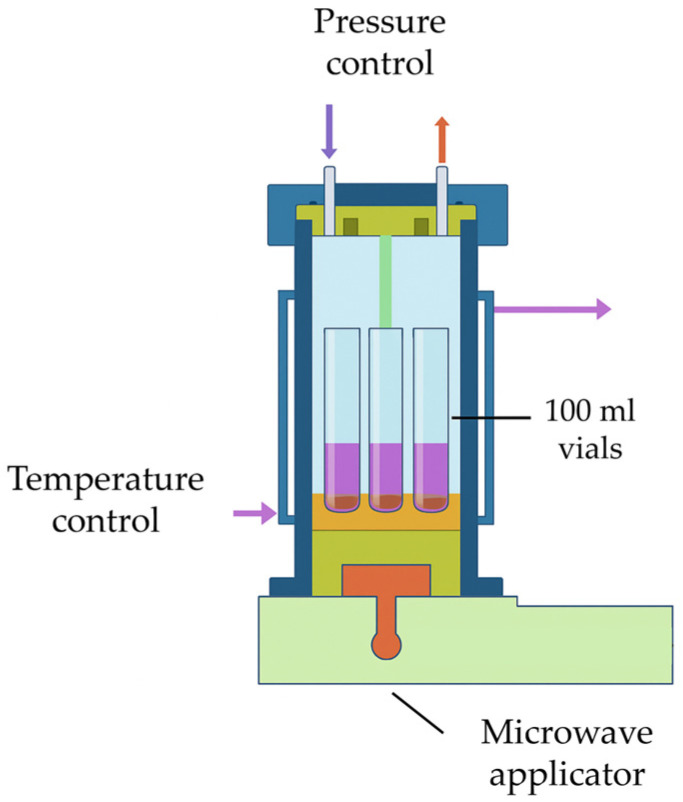
Internal configuration and key components of the MW-assisted reactor (synthWAVE).

To ensure reproducibility, all experiments followed identical sample preparation procedures, heating programs, and solvent-to-solid ratios. For the 40 mL trials, composite fragments were weighed to 3.00 ± 0.05 g and placed into 100 mL glass reaction vials sealed with Teflon caps. Each vial was positioned inside a dedicated holder, which was then placed in a 1 L Teflon-lined vessel filled with ethylene glycol (see Figure 3). The ethylene glycol acted as a thermal and dielectric bath to homogenize microwave absorption and minimize field heterogeneity. The assembled system was then inserted into the microwave reactor chamber for processing.

Temperature and pressure were controlled using the reactor’s PLC interface, with real-time monitoring of internal temperature, pressure, and applied microwave power. Heating ramps followed the programmed profiles (see Table 1), and total reaction time was defined as the duration after reaching the set temperature. After cooling to <40 °C, the reaction contents were filtered under identical conditions; fibers were washed in a 60 °C water bath for 10 min, rinsed with acetone (analytical grade, ≥99.5%), and dried at 60 °C for 4 h before weighing.

Microwave power delivered to the load was automatically modulated by the synthWAVE system to maintain the set temperature. Maximum power during a typical 230 °C program ranged between 800 and 1200 W. These operational details, together with the specified sample size, solvent volume, reagent purity, washing protocol, and data processing method, allow full reproducibility of the solvolysis procedure.

The solvent systems analyzed included glacial acetic acid + ZnCl_2_ (5 wt.%), ethylene glycol + ZnCl_2_ (5 wt.%), and urea–glycerol–choline chloride (1:1:1 molar) as a DES alternative. After identifying the most effective configuration at the 40 mL scale, the same setup was applied to the manufacturer’s composites and subsequently scaled up to 1 L with the CDW sample.

### 2.4. Analytical Methods

#### 2.4.1. Characterization of Composites

All composite samples underwent calcination to quantify their resin content. The process parameters were adjusted according to the type of reinforcing fibers and the thickness of the composite specimens, considering experimental conditions previously reported in the literature [15,16]. Notably, composites sourced from the CDW manager, which exhibited significantly lower thickness, required different thermal treatment conditions compared to those supplied by the manufacturer (see Table 2).

To determine the resin content, each composite was weighed before and after calcination. The resin fractions are reported as mean ± standard deviation from five independent replicates (*n* = 5) and were calculated as follows:

(1) $$\%Resin=\frac{m_{1}-m_{2}}{m_{1}}\times 100$$

where m_1_ (g) is the initial mass of the composite and m_2_ (g) is the mass of the remaining solid after calcination.

Knowledge of the resin and fiber content allowed for a quantitative evaluation of solvolysis efficiency across the experimental process.

To further characterize the polymeric composition of the unknown CDW material, a qualitative assessment of the functional group composition of the samples was performed using Fourier-Transform Infrared Spectroscopy (FTIR). Measurements were conducted with a Spectrum Two spectrometer (PerkinElmer, Waltham, MA, USA; 6PG10052-048) over the spectral range of 4000–450 cm^−1^. The composite surface was placed in direct contact with the UATR (Universal Attenuated Total Reflectance) sample holder (PerkinElmer, Waltham, MA, USA; 6PG10052-048-02) and the resulting infrared spectra were compared with reference databases and standard samples to determine the chemical identity of the material.

**Figure 4:**
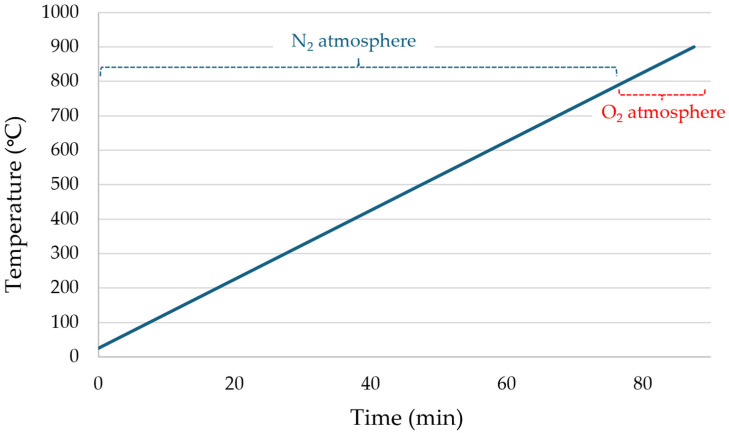
Temperature program used in the TGA.

Thermogravimetric analysis (TGA) was performed using a TGA 8000 (PerkinElmer, Waltham, MA, USA; 6PG10052-080) with a 10 °C min^−1^ heating rate under two consecutive atmospheres to separate the main thermal events (Figure 4): Stage 1 = 25–800 °C under N_2_ to evaluate volatile release and primary degradation under inert conditions, followed by Stage 2 = 800–900 °C under O_2_ to calcine the remaining organic residues (char) and thereby determine the inorganic fraction. In addition, coupled TGA–FTIR analysis (4000–625 cm^−1^) enabled gas-phase identification of degradation products.

#### 2.4.2. Characterization of Recovered Products

After each reaction, the solid and liquid phases were separated by filtration. The recovered fibers were thoroughly washed by immersion in a hot water bath (60 °C) for 10 min, followed by an acetone rinse. This process ensures the removal of degradation residues from the fiber surface. Finally, fibers were dried in an oven at 60 °C for 4 h and weighed, for the degradation yield determination.

In trials achieving ≥80% matrix degradation, scanning electron microscopy (SEM) was used to inspect the fibers for possible ZnCl_2_ residues and structural damage using an IN-SPECT-F50 instrument (FEI, Hillsboro, OR, USA). The samples were placed in an aluminum sample holder on carbon tape by cutting a set of fibers with scissors and carefully gluing them onto the tape. Subsequently, 20 nm of carbon was deposited using a carbon coater (EM ACE200, Leica Microsystems GmbH, Wetzlar, Germany) and carbon wire as a source. The fibers were then observed under high vacuum and room temperature conditions and an accelerating voltage of 10 V and a spot size of 3 were used for imaging.

For determining the chemical compositions of the observed residues on the fibers, X-ray energy dispersive spectroscopy (XR-EDS) analysis with a spot size of 4 and spot spectra were performed.

On the fibers recovered from the experiments on the manufacturer composites of known initial characteristics, mechanical tests were carried out to evaluate to what extent the fibers maintained their properties after the solvolysis process. Mechanical testing followed ASTM C1557 using a Favimat+ tensile tester (Textechno Herbert Stein GmbH & Co. KG, Mönchengladbach, Germany) [19]. Fibers were taken from both infusion and pultrusion composites to measure tensile strength, modulus, and elongation at break.

## 3. Results and Discussion

### 3.1. Material Characterization

#### 3.1.1. CDW Composite

**Figure 5:**
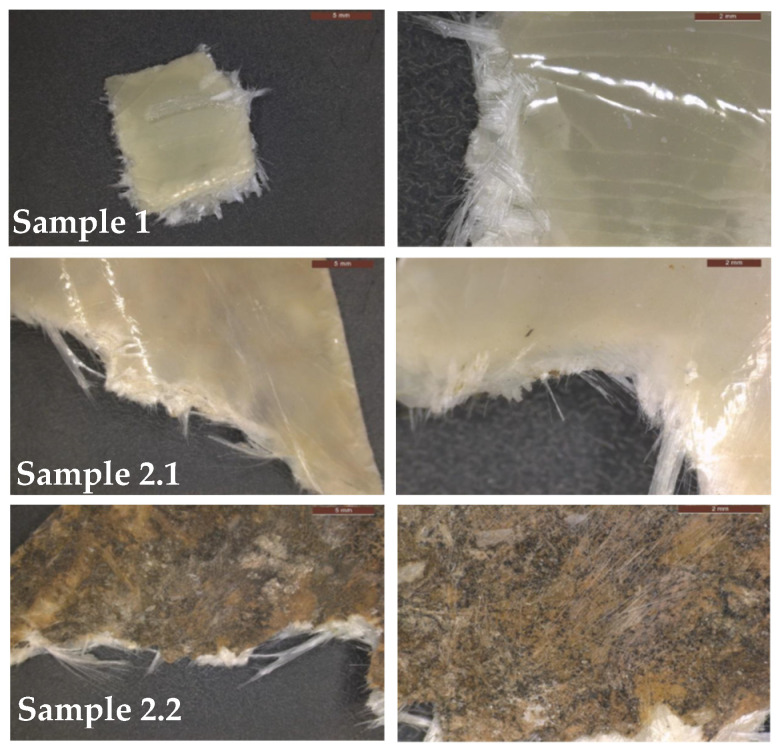
CDW samples analyzed by FTIR-TGA. Sample 1 (**top**): cleaned and cut, Sample 2.1 and Sample 2.2 (**middle** and **bottom**): non-cleaned and uncut photographed from both sides showing superficial deposits.

The compositional and thermal analysis of the CDW composite was conducted prior to solvolysis to determine the resin type and guide the selection of optimal degradation conditions. The polymeric matrix of the CDW sample was analyzed in its ‘as-received’ conditions and after superficial dust removal. Figure 5 presents an enlarged image of the analyzed materials, including clean and cut samples (Sample 1) and the non-cleaned and uncut samples (Sample 2.1 and Sample 2.2).

**Figure 6:**
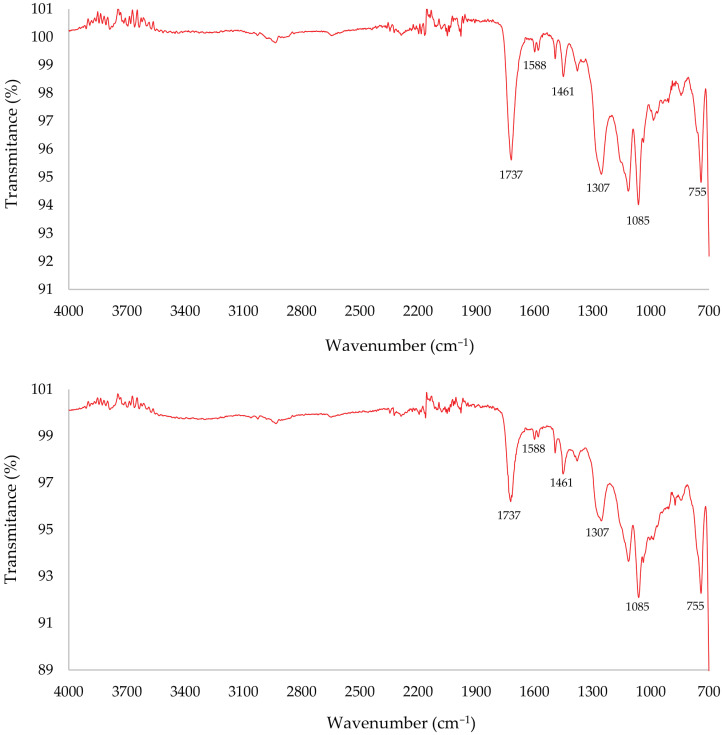
FTIR results for Sample 1 (**top**) and Sample 2 (**bottom**), showing characteristic bands of an unsaturated polyester: ester—1737 cm^−1^ (C=O) and ~1150 cm^−1^ (C–O–C); aromatic ring—1588 cm^−1^ (C=C) and 755 cm^−1^ (C–H); aliphatic—1461 cm^−1^ (CH_2_/CH); backbone unsaturation—1307 cm^−1^ (C=C).

According to their respective FTIR spectra, shown in Figure 6, no significant differences were observed between the samples, indicating that the superficial deposit did not interfere with the chemical composition analysis. The interpretation of both analyses indicates that any surface dust is either IR-inert or too thin to interfere with the polymer spectrum; therefore, the surface matrix chemistry is representative of the bulk.

The spectrum of the CDW composite exhibits a distinct absorption band at 755 cm^−1^ and a weaker one around 1000 cm^−1^, corresponding, respectively, to the –C–H bending vibrations in the 1 and 3 positions of a benzene ring and –C=CH bending associated with the isomerization of maleic anhydride to fumarate during polymerization [17]. A broad absorption cantered at approximately 1150 cm^−1^ evidences the presence of –C–O–C– stretching vibrations, characteristic of ester linkages [17,18]. The band at 1307 cm^−1^ is attributed to the –C=C– stretching typical of polyester backbones, while a moderate band at 1461 cm^−1^ reflects –C–H bending vibrations [17,20]. Minor multiple bands around 1588 cm^−1^ correspond to aromatic C=C ring stretching modes. A strong absorption band at 1737 cm^−1^ confirms the presence of ester carbonyl (–C=O) groups, corroborating the formation of a polyester resin [17,18,20]. Moreover, the disappearance of the anhydride peak at 1760 cm^−1^, originally present in maleic and phthalic anhydrides, together with the loss of the hydroxyl peak at 1373 cm^−1^ from propylene glycol, further corroborates the complete polyesterification of the resin [17,18].

**Figure 7:**
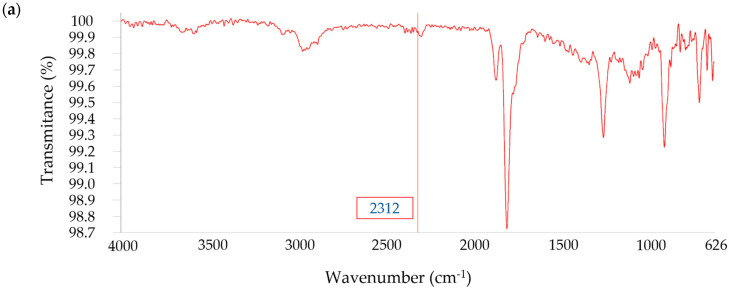

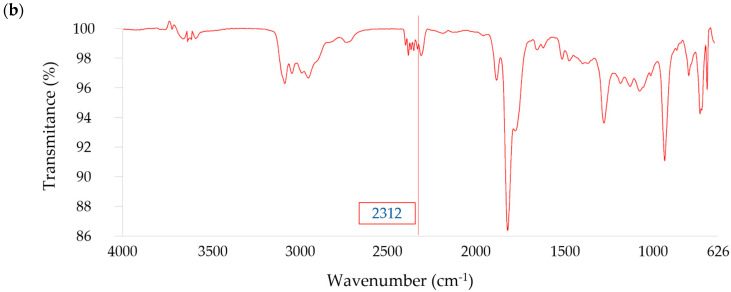
FTIR spectra of gases released during TGA at DTG peak temperatures: (**a**) 191 °C and (**b**) 367 °C. Carbonyl (C=O) 1750–1700 cm^−1^; ether (C–O) 1200–1100 cm^−1^; aliphatic (C–H) 3000–2850 cm^−1^.

When entering the spectrum in the KnowItAll database, it identifies these resins as COREZYN 316 (Hit Quality Index: 83.27), a flexible polyester resin. Thermal degradation analysis (Table 3) revealed two major weight-loss stages: 9.1 wt.% between 123 and 280 °C, associated with low-molecular-weight volatiles, and 73.3 wt.% between 280 and 475 °C, corresponding to polymer decomposition. The residual inorganic content after combustion was 2.2 wt.%. FTIR analysis of gaseous products released during TGA (Figure 7) showed identical spectra for both decomposition peaks, characterized by carbonyl and ether vibrations and aromatic groups.

The different degradation temperatures may be attributed to the molecular weight of the polymer. The spectra are characterized by the presence of various carbonyl (C=O) and ether (C–O) groups. Additionally, peaks in the fingerprint region suggest the presence of aromatic groups. The FTIR analysis of gases evolved during TGA revealed the presence of anhydrous succinic acid at 191 °C and phthalic acid monomethyl ester at 367 °C, both of which are typical decomposition products of unsaturated polyester resins containing maleic and phthalic anhydride precursors. These results further confirm the polyesteric nature of the CDW composite matrix and its degradation through ester bond cleavage. The quantification of resin and glass fiber contents (Table 4) indicated that the CDW sample contained 77.9 wt.% polyester resin and 22.1 wt.% glass fiber. Such a high resin proportion is typical of hand lay-up or low-pressure composite systems and supports the observed high degree of polymer degradation during solvolysis. These compositional data provided a reference for evaluating reaction efficiency in subsequent sections.

#### 3.1.2. Manufacturer’s Composites

The manufacturer’s samples, composed of epoxy, vinyl ester, and polyester resins reinforced with glass fiber, exhibited distinct resin-to-fiber ratios (Table 4). Epoxy pultrusion composites contained the lowest resin content (27.3 ± 1.2 wt.%), while polyester pultrusion showed the highest (80.7 ± 2.6 wt.%). These differences strongly influence solvolysis behavior, as higher resin fractions enhance solvent diffusion but also increase reaction viscosity. These contrasting resin–fiber configurations enabled a comparative assessment of solvolysis behavior, helping to elucidate the role of matrix chemistry and composite architecture in determining reaction yield and fiber preservation.

### 3.2. Solvolysis Results

#### 3.2.1. Optimization and Parameter Influence

**Figure 8:**
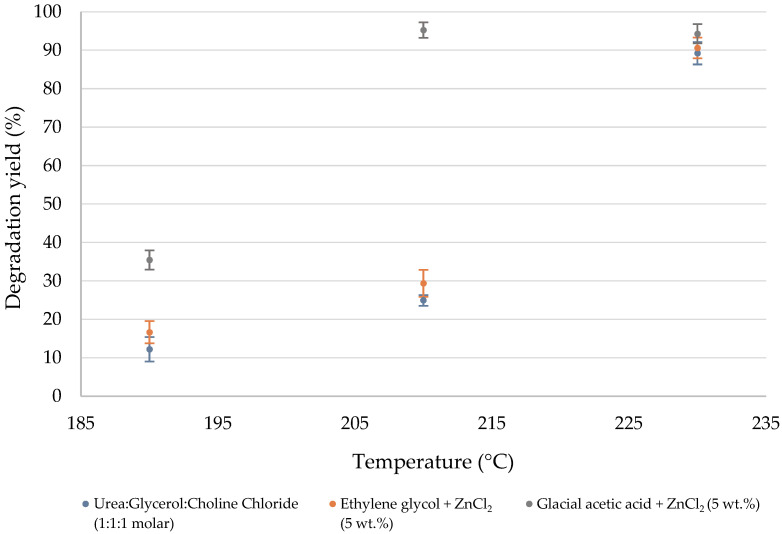
Degradation yield of the CDW sample under different experimental conditions.

The influence of temperature, reaction time, and solvent system on resin degradation was systematically evaluated at the 3 g batch scale using CDW samples (Figure 8). A set of experimental conditions was tested by varying temperature and reaction time and comparing solvent systems to identify their impact on the degradation yield. Degradation yields were calculated according to the following:

(2) $$\%Degradation\;yield=\frac{m_{1}-m_{2}}{m_{1}\cdot \%Resin}\times 100$$

where m_1_ (g) is the initial mass of the composite, m_2_ (g) is the mass of the remaining solid after solvolysis, and %Resin is the original resin content of each composite, calculated according Equation (1). Results were averaged across five independent replicates (*n* = 5).

The experimental results confirm that temperature is the key parameter governing the depolymerization process. In the urea- and ethylene glycol-based systems, a substantial degradation is only achieved under the more energetic program (Program 1). In contrast, the glacial acetic acid + ZnCl_2_ (5 wt.%) medium, with a lower activation energy, attains higher degradation even at lower temperatures (Program 2), as once the activation energy is surpassed, the longer reaction time allows the reaction to proceed further, resulting in slightly higher yields.

**Figure 9:**
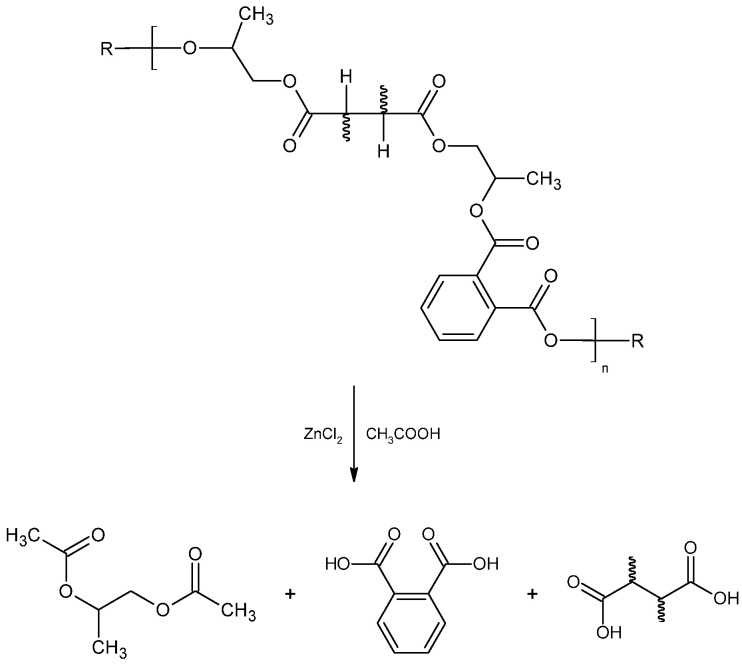
Solvolysis mechanism of polyester degradation in an acetic acid/ZnCl_2_ reaction medium.

Overall, the acetic acid system consistently achieved the highest degradation levels, up to 95%, outperforming ethylene glycol and the DES mixture. Its superior performance is attributed to the high swelling capacity of acetic acid, which enhances ZnCl_2_ diffusion and activation of ester bonds [21], as illustrated by the proposed reaction mechanism in Figure 9.

In contrast, the DES and ethylene glycol systems were limited by their lower polarity and reduced ability to penetrate the polymer network, resulting in incomplete depolymerization of the polyester matrix. This comparison highlights the key role of solvent–polymer interactions and catalytic activation in determining overall solvolysis efficiency, underscoring the suitability of acetic acid + ZnCl_2_ as the most effective reaction medium for subsequent optimization and scale-up.

It should be noted that the data presented here evaluate the macroscopic solvolysis performance of DESs under microwave heating, not the underlying dielectric or molecular-scale interactions. Further work is required to elucidate the fundamental MW–DES mechanisms.

Comparing heating programs, the highest temperature profile (Program 1, 230 °C, 120 min) yielded optimal results for all solvents. However, in acetic acid + ZnCl_2_, Program 2 (210 °C, 160 min) achieved nearly identical efficiency, suggesting that this system operates effectively at reduced activation energy. This highlights the potential for energy savings during scale-up. In contrast, for DES and ethylene glycol systems, increasing time without temperature elevation did not improve yield, confirming that these reactions are kinetically limited and possess higher activation energies. This behavior reinforces the importance of optimizing both thermal input and solvent reactivity to balance efficiency and sustainability in microwave-assisted solvolysis processes.

**Figure 10:**
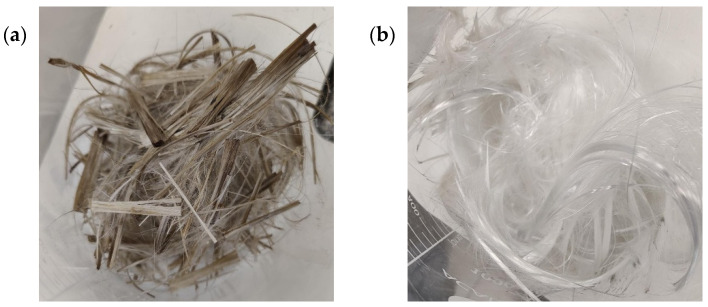

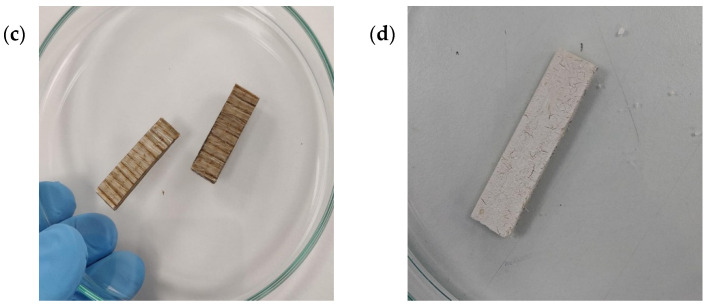
Reclaimed fibers after the solvolysis process: (**a**) epoxy infusion (EP-IN), (**b**) epoxy pultrusion (EP-PU), (**c**) vinyl ester infusion (VE-IN) and (**d**) polyester pultrusion (EST-PU).

To further evaluate the generality of the optimized conditions, additional experiments were carried out using the manufacturer’s composite samples containing different polymeric matrices. Each test was conducted at the 3 g batch scale under the same optimal parameters (230 °C, 120 min, glacial acetic acid + ZnCl_2_ 5 wt.%). Degradation yields, summarized in Table 5, are reported as mean ± standard deviation from five independent replicates (*n* = 5) and were calculated according to Equation (2). The results demonstrate significant variability in degradation yield depending on the resin type and manufacturing process. The appearance of the samples after the reaction is shown in Figure 10.

These findings reveal that epoxy-based composites, particularly those produced by pultrusion, exhibited the highest degradation efficiencies, likely due to the high cross-link density and uniform matrix structure that favor solvent penetration and catalytic activation. Vinyl ester and polyester systems, on the other hand, showed markedly lower reactivity under the same conditions, reflecting differences in ester bond accessibility and network stability. Overall, these results confirm the applicability of the optimized solvolysis parameters across multiple resin chemistries, while highlighting the influence of manufacturing method on process efficiency and fiber recovery potential.

#### 3.2.2. Upscaling Performance and Solvent Recovery

**Figure 11:**
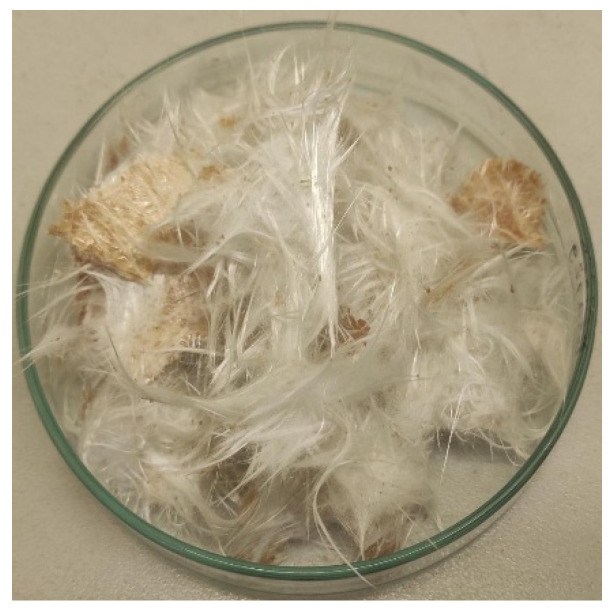
Reclaimed fibers from CDW sample solvolysis.

To assess scalability, the most promising configuration (glacial acetic acid + ZnCl_2_, 230 °C, 120 min) was applied in a 1 L reactor processing 45 g of CDW composite and 600 mL solvent. The reclaimed fibers are shown in Figure 11. Larger sample pieces than those used in the previous section were used to enable mechanical testing, to determine the damage the fibers had been exposed to during the solvolysis process, as these tests require a minimum fiber length of 5 cm to be carried out properly. Due to physical constraints of the reactor design, stirring could not be applied during these experiments.

When scaling up the system at the same solvent-to-solid ratio (~13.3 mL/g), the conversion decreased from 94% to 78% (Table 6). This may be explained by several factors. For instance, the use of larger fiber pieces at a larger scale raises the diffusion time, reducing the effectiveness factor and slowing depolymerization. In parallel, the greater load and fill height likely produce less uniform microwave fields and lower average temperatures in parts of the bed. Although global solvent saturation is unlikely at a fixed mL/g ratio, local product build-up and viscosity increases near particle surfaces can thicken boundary layers without active agitation, further depressing external mass transfer. These observations suggest that restoring efficiency at scale should focus on transport and heating uniformity. This may be achieved by narrowing and reducing fragment size, introducing mechanical stirring or recirculation with baffles, limiting fill height (or using wider, shallower reactors), and employing microwave susceptors or mode stirring to homogenize the temperature.

Given that the main objective of the study is to make the valorization process as environmentally sound as possible, solvent recovery was also addressed. The solvent recovery tests confirmed the environmental viability of the process. Vacuum distillation recovered 85% of the initial acetic acid, with ICP/OES analysis showing negligible zinc contamination. The recovered solvent maintained its reactivity when reused for epoxy pultrusion composite solvolysis, achieving comparable degradation efficiency. This demonstrates that the process can operate in a semi-closed loop with minimal reagent losses, aligning with circular economy principles.

### 3.3. Recovered Fiber Analysis

#### 3.3.1. Morphology (SEM/EDS)

**Figure 12:**
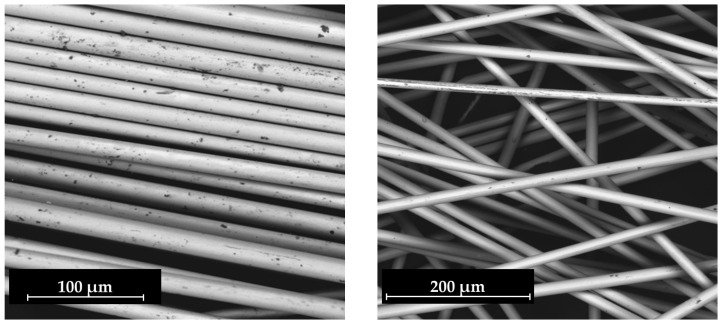
SEM images of reclaimed fibers: EP-IN GF (**left**) and EP-PU GF (**right**).

An SEM analysis was performed on the reclaimed fibers obtained from the solvolysis with glacial acetic acid + ZnCl_2_ carried out under Program 1, selecting samples that exhibited a degradation yield above 80% (epoxy-based composites). As shown in Figure 12, reclaimed fibers from infusion-based composites appeared more closely packed, consistent with lower degradation yields, whereas those from the pultruded composites exhibited clean surfaces and were fully separated. EDS analysis confirmed the absence of ZnCl_2_ residues, indicating effective post-washing and good solvent compatibility with fiber preservation.

These findings validate that the solvolysis conditions applied were sufficiently selective to decompose the polymeric matrix without compromising the glass fiber structure. The preserved fiber morphology observed after treatment indicates that microwave-assisted heating promotes uniform energy distribution, enabling effective resin removal while avoiding localized overheating or etching damage. Furthermore, the absence of inorganic or catalytic residues detected by EDS demonstrates that ZnCl_2_ remained confined to the liquid phase and was efficiently removed during washing. Overall, the morphological and elemental analyses confirm that the process yields clean, undamaged fibers suitable for subsequent mechanical testing and potential reuse in composite manufacturing.

#### 3.3.2. Mechanical Properties

The mechanical properties of the recycled fibers were evaluated for both infusion and pultrusion epoxy composites, with nominal fiber diameters of 17 µm and 24 µm, respectively. These two systems were selected owing to their favorable matrix reactivity in glacial acetic acid + ZnCl_2_ (5 wt.%), as indicated in Table 7, and to enable a direct comparison with virgin fibers to quantify the loss of performance associated with thermal–chemical exposure during solvolysis. To minimize artifacts from residual polymers and following the same criterion applied in the superficial analysis, only fibers recovered from trials achieving over 80% matrix degradation were subjected to tensile testing.

Regarding the reclaimed fibers, two diameters were consistently identified corresponding to their use in infusion or pultrusion processes. This distinction reflects the manufacturing requirements of each technology: pultrusion composites are generally designed to withstand higher mechanical loads and thus adopt thicker fiber diameters, whereas infusion fabrics employ finer filaments to promote impregnation and drapability. The resulting geometry therefore mirrors the process-specific demands placed on each composite architecture.

Table 7 summarizes the experimental conditions under which the epoxy infusion (EP-IN GF) and epoxy pultrusion (EP-PU GF) fibers used for mechanical tests were obtained at 230 °C, including trials with fresh and recovered acetic acid + ZnCl_2_ media.

Mechanical characterization (see Table 8) revealed a partial loss of tensile performance relative to virgin fibers, with behavior dependent on manufacturing route and fiber diameter. For infusion-based epoxy fibers (EP-IN GF, 17 µm), tensile strength decreased by ~30% and elongation at break by ~45%, which is consistent with sizing removal and the cumulative thermal–chemical stress endured during solvolysis. In contrast, pultrusion-based epoxy fibers (EP-PU GF, 24 µm) retained most of their properties, exhibiting 16–19% reductions in tensile strength while modulus values remained broadly comparable to the reference. Notably, no systematic diameter loss was detected in the pultrusion fibers, suggesting that the sizing system in this product was less reactive under the tested conditions, thereby moderating strength degradation relative to the infusion case.

Importantly, the recovered solvent trial (Experiment VI) produced fibers whose tensile response fell within the same order of magnitude as those obtained with fresh solvent, indicating that solvent reuse did not measurably compromise fiber performance. Taken together, these findings show that (i) manufacturing route and fiber diameter are key determinants of mechanical retention after solvolysis, and (ii) the acetic acid + ZnCl_2_ system can be operated in a semi-closed loop without evident detriment to fiber quality, provided that high matrix-degradation levels are achieved prior to testing.

Apparent increases in the modulus of some recycled glass fibers are linked to the stress–strain reduction when machine/grip compliance is not fully corrected and the filament area is estimated from the measured diameter. As presented in Table 8, E varied approximately with 1/d^2^. After normalizing the recycled data to the reference diameter, the EP-PU moduli converge to ~63 GPa (±1 GPa); thus, there was no significant intrinsic change. The EP-IN batch ‘I’ remains ~12% higher even after normalization, which can be attributed to genuine stiffening from thermal conditioning (structural relaxation and de-hydration of the glass) and possibly reduced grip slippage on de-sized fibers. This is consistent with the concurrent drop in strength and elongation.

Regarding secondary application of the recovered fibers, the observed tensile strength and modulus shifts are largely compatible with secondary, stiffness-driven uses where design and safety factors already provide a margin. Recycled fibers from the EP-PU series show strength reductions that are mostly modest (~17–19% in II, III, and VI) or even gains (IV: +16%, V: +4.5%), with moduli clustering near the reference once diameter effects are accounted for (−6 to +12%). Such variations are typically tolerable in non-critical laminates, short-fiber compounds, and semi-structural panels, where service stresses are a small fraction of ultimate strength and stiffness governs deflection, acoustic response, or dimensional stability. Even the EP-IN I case, which shows a larger strength drop (~31%), is accompanied by a substantial modulus increase (~25%), meaning that for stiffness-limited components (i.e., covers, housings, interior panels, spacer profiles, and cable trays), the functional performance can be preserved by minor adjustments in fiber content, lay-up, or section thickness without compromising safety. In practice, typical proof load criteria and safety factors (often ≥2) absorb 10–20% degradations in tensile capacity without redesign. When losses approach ~30%, straightforward compensations, like slightly higher fiber volume fraction, improved sizing/adhesion, or local thickness increases, restore margins at minimal cost. Consequently, the measured property changes fall within acceptable limits for a wide range of secondary applications, provided that parts are not intended for primary load paths, pressure containment, or high-cycle fatigue-critical duty, where dedicated qualification would still be required.

## 4. Potential for Impact on Sustainability and Circularity

The MW-assisted chemical recycling strategy presented in this work represents a step forward in the sustainable management of fiber-reinforced composites (FRPCs) derived from construction and demolition waste (CDW). By integrating green solvents and energy-efficient microwave heating, the method effectively addresses the environmental and economic limitations of conventional recycling and disposal routes. The approach embodies the principles of the circular economy by combining material recovery, solvent recirculation, and reduced energy demand within a single closed-loop system.

To quantify the energy and carbon savings associated with the MW-assisted solvolysis process, the energy demand was estimated from the average microwave power delivered to the load (700–900 W, measured by the internal power controller) multiplied by the effective reaction time at a set temperature (40–120 min depending on the program). This corresponds to an energy input of approximately 7–9 MJ per kilogram of composite processed, assuming a solid-to-solvent ratio of 3 g to 40 mL and an 80% volumetric heating efficiency, consistent with reported MW-assisted chemical processes. For comparison, typical thermochemical recycling technologies, such as pyrolysis, operate at 400–700 °C with reported energy requirements of around 22 MJ/kg of composite [22,23,24]. The energy saving of the present method was therefore calculated as follows:

Energy saved (MJ/kg) = 22 MJ/kg − (7–9 MJ/kg) = 13–15 MJ/kg

The avoided greenhouse gas emissions were estimated using the EU-average electricity emission factor (0.095 kg CO_2_ eq/MJ) [25], yielding the following:

CO_2_ saved (kg CO_2_ eq/kg) = (13–15 MJ/kg) × 0.095 = 1.24–1.43 kg CO_2_ eq/kg

This approach provides a transparent and reproducible basis for the sustainability claims made, confirming that the proposed MW-assisted process operates with substantially lower energy input and lower environmental burden than conventional thermochemical routes.

The economic implications of these energy and solvent efficiencies are also noteworthy. Reported operational costs for pyrolysis and glycolysis processes range between 1.2 and 1.8 EUR/kg of treated composite, driven largely by high thermal energy requirements and solvent losses [5,25]. In contrast, the mild conditions and solvent recirculation demonstrated in this study suggest a potential operating cost near 0.8 EUR/kg, assuming proportional reductions in energy input and solvent replacement needs. The solvent recovery yield achieved by vacuum distillation reached 85%, while ICP/OES analysis confirmed negligible zinc contamination, indicating the feasibility of repeated solvent reuse. The ZnCl_2_ catalyst can also be effectively recovered by simple precipitation and filtration, with a recyclability above 95%, consistent with previously reported acetic acid–ZnCl_2_ systems [6]. These combined factors significantly reduce consumable costs and chemical waste generation, aligning the process with the green chemistry principles of resource efficiency.

A comparative evaluation of other reported green recycling methods further highlights the advantages of the proposed process. Typical DES- and IL-based solvolysis systems operate at 150–250 °C, with reaction times exceeding 4 h and energy demands of 12–20 MJ/kg, achieving degradation yields of 80–90% and fiber strength retention of 70–80% [6,7,8,9]. Hydrothermal processes achieve similar degradation efficiencies (85–95%) but require high-pressure water environments and comparable energy inputs (15–25 MJ/kg) [26,27]. Recently, pyridine-based solvolysis systems have demonstrated remarkable results, achieving over 90% degradation and up to 93% fiber strength retention, though they still rely on demanding conditions, operating at 250 °C for about 2 h [28]. In contrast, the present MW-assisted process reached over 90% degradation yield with 80–85% tensile strength retention after only 40 min at 230 °C, while operating at significantly lower energy consumption (7–9 MJ/kg). The rapid, uniform heating achieved by microwave irradiation enhances depolymerization kinetics without compromising fiber quality, confirming the superior energy-to-performance ratio of this approach relative to other emerging technologies.

The sustainability performance of the developed process is reinforced by several key indicators. The solvent recovery yield (85%) and ZnCl_2_ recyclability (95%) ensure that reagent circulation losses are minimal, while fiber recovery efficiency exceeded 95% in small-scale trials and remained above 78% in scaled-up experiments. The reuse of recovered solvent produced comparable degradation yields, verifying that the system can function in a semi-closed operational loop. When normalized per kilogram of recycled FRPC, the combined energy and material savings correspond to an estimated of around 55% reduction in process-related environmental impacts relative to conventional thermal recycling.

Beyond these quantitative indicators, the process contributes qualitatively to circularity by extending the useful life of recovered glass fibers through reintegration into secondary manufacturing applications. The recovered fibers retained over 80% of their original tensile strength, enabling potential reuse in non-structural composite, insulation, or textile applications. Given that the production of virgin glass fibers emits approximately 1.2 kg CO_2_ equation per kilogram, each kilogram of fiber recovered through this method represents an equivalent avoided emission [29]. This not only offsets part of the process-related carbon footprint but also reduces demand for primary raw materials, strengthening the overall environmental balance of the recycling loop.

Overall, the MW-assisted chemical recycling strategy demonstrates measurable energy and carbon savings, reagent recirculation efficiency, and material recovery performance superior to those of conventional techniques. Its compatibility with green solvents and scalability under moderate operating conditions supports its potential for industrial scalability and integration within regional CDW valorization frameworks. Future work will involve quantitative life-cycle and techno-economic analyses to validate these estimates and provide a robust evidence base for the environmental and economic viability of the process at commercial scale.

## 5. Conclusions

The present study demonstrates the technical feasibility and environmental benefits of an MW-assisted solvolysis process using glacial acetic acid + ZnCl_2_ (5 wt.%) for the recycling of fiber-reinforced polymer composites derived from both post-consumer (CDW) and pre-consumer sources, while DES- and ethylene glycol-based mixtures showed limited depolymerization efficiency under microwave heating compared with acetic acid-based media.

The proposed approach achieved resin degradation efficiencies up to 95% under mild operating conditions (230 °C, 120 min), while maintaining over 80% fiber tensile strength retention. These results confirm that the process enables efficient depolymerization of polyester and epoxy matrices, producing recovered fibers with morphology and mechanical properties suitable for reuse in secondary composite manufacturing. The experimental results revealed that epoxy pultrusion composites achieved the highest degradation yield (97.92 ± 0.3%) and fiber quality retention, whereas vinyl ester and polyester matrices exhibited lower reactivity under equivalent conditions. The integrity of the recovered fibers was preserved, with minimal surface alteration and no ZnCl_2_ residues detected by EDS, validating the selectivity of the acetic acid-based solvolysis system.

The process also exhibited notable improvements compared to conventional thermochemical routes, with an estimated energy demand of 7–9 MJ/kg of composite treated (~60–70% lower than typical pyrolysis/glycolysis values of ~22 MJ/kg), corresponding to energy savings of 13–15 MJ/kg and avoided emissions of 1.24–1.43 kg CO_2_ equation per kg of FRPC processed. Solvent recovery reached 85% and ZnCl_2_ recyclability exceeded 95%, enabling closed-loop operation. From a techno-economic standpoint, the operating cost is estimated at ~0.8 EUR/kg of composite, compared with 1.2–1.8 EUR/kg for pyrolysis-based recovery, driven by lower energy input and reagent reuse.

In sum, this research establishes microwave-assisted solvolysis as achieving quantitative improvements in degradation yield, fiber retention, and energy efficiency, while enabling solvent and catalyst reuse. Future work will focus on elucidating the fundamental microwave–solvent interaction mechanisms, scaling the technology to continuous operation, and performing full life-cycle and techno-economic assessments to validate the long-term environmental and financial benefits of this circular recycling strategy.

## Figures and Tables

**Table 1:** Design of experiments. Matrix: FRPC from CDW manager/volume: 40 mL.

Program	Temperature (°C)	Pressure (bar)	Agitation (rpm)	Time (min)
1	230	30	150	120
2	210	30	150	160
3	190	40	150	120

**Table 2:** Calcination programs by origin.

Composite	Origin	Calcination Program	Average Thickness
Glass fiber in unknown polymeric resin	CDW manager	T_i_: 15 °C	2 mm
		T_f_: 500 °C	
		Ramp: 20 °C/min	
		t_calcination_: 180 min	
Glass fiber in epoxy, vinyl ester, and polyester resin	Manufacturer	T_i_: 15 °C	6 mm
		T_f_: 800 °C	
		Ramp: 20 °C/min	
		t_calcination_: 120 min	

**Table 3:** TGA results for the characterization of CDW sample.

Temperature Range (°C)	Temperature Peak (°C)	Weight Loss (wt.%)
123.5–280.5	191.4	9.11
280.5–475.6	366.9	73.28
800	-	15.4
Inorganic ashes	-	2.21

**Table 4:** Calculation of resin and glass fiber percentages from calcination results.

Sample Supplier	Resin/Manufacturing Process	% of Resin
CDW manager	Polyester/Unknown	77.9 ± 2.3
Manufacturer	Epoxy/Infusion	35.8 ± 1.5
Manufacturer	Epoxy/Pultrusion	27.3 ± 1.2
Manufacturer	Vinyl ester/Infusion	29.1 ± 1.4
Manufacturer	Polyester/Pultrusion	80.7 ± 2.6

**Table 5:** Degradation yield of the manufacturer’s samples under 230 °C for 120 min in glacial acetic acid + ZnCl_2_ (5 wt.%).

Sample Matrix	Manufacturing Process	Nomenclature	Degradation Yield (%)
Epoxy	Infusion	EP-IN GF	81.60 ± 0.7
Epoxy	Pultrusion	EP-PU GF	97.92 ± 0.3
Vinyl ester	Infusion	VE-IN GF	51.50 ± 2.4
Polyester	Pultrusion	EST-PU GF	n.d. *

* Not detected.

**Table 6:** Experimental conditions of the up-scaled system for the CDW sample.

Sample	Reaction Solvent	Temperature (°C)	Time (min)	Degradation Yield (%)
CDW’s sample	Glacial acetic acid + ZnCl_2_ (5 wt.%)	230	120	78.41 ± 1.6

**Table 7:** Experimental conditions at which epoxy infusion and epoxy pultrusion glass fibers employed in the mechanical tests were obtained (T: 230 °C).

Solvent	Experiment	Fiber	Time (min)	Degradation Yield (%)
Glacial acetic acid + ZnCl_2_ (5 wt.%)	I	EP-IN GF	120	81.60 ± 0.7
	II	EP-PU GF	120	97.92 ± 0.3
	III	EP-PU GF	80	99.91 ± 0.1
	IV	EP-PU GF	60	99.35 ± 0.2
	V	EP-PU GF	40	98.71 ± 0.4
Recovered glacial acetic acid + ZnCl_2_ (5 wt.%)	VI	EP-PU GF	40	96.78 ± 0.5

**Table 8:** Single-fiber tensile test results.

ID	Experiment	Diameter (µm)	Tensile Strength (MPa)	Modulus (GPa)	Elongation at Break (%)
EP-IN GF	Reference	17.76 ± 1.38	1836.86 ± 190.69	55.25 ± 2.83	3.3 ± 0.4
	I (Recycled)	16.81 ± 1.79	1258.16 ± 269.57	69.32 ± 5.98	1.8 ± 0.4
EP-PU GF	Reference	24.4 ± 1.03	1798.67 ± 261.38	63.27 ± 3.64	2.8 ± 0.4
	II (Recycled)	23.41 ± 1.65	1485.8 ± 474.35	70.93 ± 4.76	2.1 ± 0.7
	III (Recycled)	24.99 ± 1.68	1455.64 ± 225.65	60.47 ± 4.49	2.4 ± 0.4
	IV (Recycled)	24.39 ± 1.57	2086.59 ± 240.37	63.38 ± 4.39	3.3 ± 0.5
	V (Recycled)	24.07 ± 1.16	1879.59 ± 130.6	64.57 ± 5.08	2.9 ± 0.2
	VI (Recycled)	25.34 ± 1.91	1463.85 ± 314.09	59.33 ± 3.07	2.5 ± 0.5

## Data Availability

Data will be made available on request.

## Abbreviations

The following abbreviations are used in this manuscript:

FRPC	Fiber-Reinforced Polymer Composite
CDW	Construction and Demolition Waste
MW	Microwave
IL	Ionic Liquid
DES	Deep Eutectic Solvent
SEM	Scanning Electron Microscopy
ICP/OES	Inductively Coupled Plasma Optical Emission Spectroscopy
CCD	Charge Coupled Device
FFT	Fast Fourier Transform
FTIR	Fourier-Transform Infrared Spectroscopy
UATR	Universal Attenuated Total Reflectance
TGA	Thermogravimetric Analysis
XR-EDS	X-ray Energy Dispersive Spectroscopy
EP-IN GF	Epoxy resin (infusion)/Glass Fiber
EP-PU GF	Epoxy resin (pultrusion)/Glass Fiber
VE-IN GF	Vinyl ester resin (infusion)/Glass Fiber
EST-PU GF	Polyester resin (pultrusion)/Glass Fiber
